# A novel approach to peer support for academic researchers

**DOI:** 10.1093/occmed/kqae091

**Published:** 2024-10-19

**Authors:** S Khodabakhsh, C Hoffmann, S Sauchelli, G Shi, A Mitchell

**Affiliations:** Faculty of Social Sciences and Law, University of Bristol, Bristol BS8 1TX, UK; National Institute for Health and Care Research Bristol Biomedical Research Centre, University Hospitals Bristol and Weston NHS Foundation Trust and the University of Bristol, Bristol BS8 2BN, UK; National Institute for Health and Care Research Bristol Biomedical Research Centre, University Hospitals Bristol and Weston NHS Foundation Trust and the University of Bristol, Bristol BS8 2BN, UK; School of Cellular and Molecular Medicine, University of Bristol, Bristol BS8 1TD, UK; National Institute for Health and Care Research Bristol Biomedical Research Centre, University Hospitals Bristol and Weston NHS Foundation Trust and the University of Bristol, Bristol BS8 2BN, UK; School of Healthcare Sciences, College of Biomedical and Life Sciences, Cardiff University, Cardiff CF14 4XN, UK

## Abstract

**Background:**

Academic researchers experience high levels of stress, isolation and loneliness, which compromise their well-being. There is a particular need to address these issues amongst early career and postgraduate research staff. ‘Spaces for Listening’ is an initiative to increase active listening and provide peer support.

**Aims:**

To assess the feasibility, acceptability and potential impacts of Spaces for Listening in an academic setting.

**Methods:**

Early career and postgraduate researchers from a large university in the UK were invited to attend ‘Academic Spaces for Listening’ (ASfL). Five ASfL sessions (including in-person and Chinese language) were held. A mixed-methods study using online survey and in-depth interviews was conducted. Quantitative data were analysed using descriptive statistics, and qualitative data were analysed using principles of thematic analysis. The qualitative and quantitative findings were integrated at the interpretation phase.

**Results:**

A total of 25 participants attended an ASfL session, 22 of them completed the survey and 6 participants participated in semi-structured interviews. Participants were very satisfied with the session content (68%, *n* = 15), organization (68%, *n* = 15) and delivery (68%, *n* = 15). Four themes were identified from qualitative analysis: (i) the ‘Academic Spaces for Listening’ (ASfL) experience; (2) impact of ASfL; (3) potential challenges of ASfL and (4) ASfL in the future. Interpersonal connectedness was an important outcome for participants during the session. Participants showed interest in the future of ASfL.

**Conclusions:**

Implementing Spaces for Listening in an academic setting is feasible and was well received by participants. The initiative may fill a gap in the social interactions amongst academic researchers.

## Teaser

This study explores the feasibility, acceptability and potential impacts of ‘Spaces for Listening’ in an academic setting. Findings suggest that implementing ‘Spaces for Listening’ is feasible and well received by participants, with the potential to improve active listening and relational awareness within universities.

## Introduction

Over the past few decades, the university sector has experienced wide-ranging and accelerated transformation [1]. There has been a rapid growth in student numbers, pressure on both early career and senior staff to secure limited research funding and the use of performance metrics (e.g. league tables) to determine the allocation of internal research funds [2]. These changes are aimed to grow revenue and productivity but can also lead to increased workload and performance pressures on academic researchers, which may negatively impact their work-life balance and well-being [3,4].

The transition to hybrid working (combination of working remotely and in-office) during coronavirus disease 2019 (COVID-19) increased the likelihood of isolation [5,6]. Academic researchers reported reluctance to disclose experienced difficulties and dissatisfaction, perceived lack of support and a negative impact on physical as well as mental health [6]. Early-career researchers (ECRs) and postgraduate researchers (PGRs) are particularly affected, reporting decreased well-being, isolation and loneliness [7,8].

There is an ongoing need to address well-being, isolation and loneliness amongst researchers. Solutions must be flexible and simple to meet the needs of remote working and to minimize the burden for those already affected by lower morale. Peer support (online and in-person) initiatives can be a simple way to address isolation and enhance well-being [9,10]. However, the evidence on the impact of peer support on overall well-being in academic settings is mostly based on support for young adults and the early years of university life [11]. The literature on academic researchers is limited, and little is known about feasible and acceptable solutions that can offer support to PGRs and ECRs.

An example of a novel peer support initiative is ‘Spaces for Listening’ [12]. ‘Spaces for Listening’ is a small group session for attendees to share their thoughts and feelings and listen to others without interruption. The initiative originated from experiences of isolation during the COVID-19 pandemic. It aims to improve communication skills and active listening and provide a safe and non-judgmental space for participants to speak without interruption. ‘Spaces for Listening’ has been adopted within large healthcare institutions in the UK [13]. Distinct advantages are the initiative’s flexibility and suitability to a multitude of settings (online and in-person). Sessions can be self-initiated and managed within the organization, which require minimal resources and do not require pre-existing infrastructure or staff training.

**Key learning points**

**What is already known about this subject:**

- Academic researchers experience high levels of stress and loneliness, and this affects their well-being.
- Spaces for Listening is an initiative promoting active listening and communication.

**What this study adds:**

- This study is the first to explore the application of Spaces for Listening and evaluate its potential impact.
- Spaces for Listening is a feasible initiative for peer support with the potential to improve well-being of academic researchers.

**What impact this may have on practice or policy:**

- Universities could adopt Spaces for Listening as an approach to improve communication, relational awareness and well-being amongst academic researchers and wider members of the university.

Early feedbacks on ‘Spaces for Listening’ show that participants feel they are being listened to, build meaningful connections and experienced active support [14]. Therefore, the aim of this study was to pilot ‘Spaces for Listening’ in academic settings, referred to hereon as ‘Academic Spaces for Listening’ (ASfL), and to evaluate the feasibility, acceptability and potential impacts of this initiative amongst PGRs and ECRs.

## Methods

This study employed a mixed-method design to evaluate the feasibility, acceptability and potential impacts of ASfL.

The study was conducted alongside pilot implementation of ASfL sessions amongst PGR and ECR staff at the University of Bristol (UoB) between March and July 2022.

All PGRs and ECRs at the UoB were eligible to take part. This cohort was chosen because it allows pilot testing on a smaller group before potential roll-out to a wider staff base. The University uses the term PGR to mean any student enrolled in Doctoral studies. Dedicated PGR mailing lists exist to target communication to this group. There are, however, no definitions or criteria to identify ECRs at the University. The study therefore relied on self-identification, and communication channels were chosen based on assumptions that their target groups include or are primarily ECRs. Invitations to attend ASfL sessions were sent via university bulletins/newsletters, email distribution through relevant mailing lists and personal networks. Participants expressed interest by contacting a member of the research team and were offered a date and time to attend an ASfL session.

Details about how ASfL sessions were conducted can be found in Table 1 (available as Supplementary data at Occupational Medicine Online) and are briefly outlined later.

ASfL sessions involved three rounds of peer-led, pre-defined questions for which participants took turns to answer. The three pre-defined questions were ‘How are you and what is on your mind right now?’; Share your reflections and feelings now, and in the light of what you have heard in Round 1” and ‘Share one thing you might like to take forwards, and also to offer some appreciation to any particular thoughts or any aspects of the call that have resonated with you’.

Each participant, including a nominated facilitator, was given 2 minutes to speak uninterrupted, in each round. Each session hosted up to eight participants (including facilitators, as stipulated by the session developers). Content discussed during the sessions was confidential, and no data were collected during the sessions.

Data collection was performed after each ASfL session and involved (i) an online survey (quantitative data) and (ii) semi-structured interviews (qualitative data).

All participants received a link to an online survey following their participation in an ASfL session. The survey was used to collect information about participants’ socio-demographic characteristics and experience of the ASfL session. The survey was designed by the research team in English language (Table 1, available as Supplementary data at Occupational Medicine Online), delivered using Microsoft Forms and analysed using Microsoft Forms analytics to report descriptive statistics. A five-point Likert scale (with endpoints ‘very much agree’ and ‘very much disagree’) was used to assess participants’ satisfaction and attitudes relating to the session [15]. At the end of the survey, participants were invited to provide their email address to express their interest in taking part in an interview. Email addresses were stored separately to maintain anonymity.

Semi-structured interviews were conducted using a topic guide (Table 2, available as Supplementary data at Occupational Medicine Online), which was developed based on the research questions and through team discussion. The interview questions were designed to explore survey answers in greater depth and to obtain understanding of why and how participants held certain views. Interviews included questions focussing on participants experience during the sessions, their reflection following their attendance and how ASfL could fit in an academic setting.

The interviews were conducted either in-person or online using Microsoft Teams. Audio-recorded data were transcribed either by an external transcription service or with the help of automatic transcription functions in Microsoft Teams.

Interview data were analysed using principles of inductive thematic analysis [16]. We followed the recommended steps suggested by Braun and Clark [16], including (i) familiarizing with the data, (ii) generating initial codes, (iii) generating initial themes, (iv) reviewing themes, (v) defining and naming themes and (vi) writing a report. Initial codes were developed by the research team to triangulate interpretations of the data. First, S.K. and S.S. independently coded two transcripts and then met to compare and discuss. Second, C.H. coded two further transcripts using the initial codes. Third, codes, initial themes and general impressions of the data were discussed within the team and amendments to the codes were made where necessary. Finally, the remaining transcripts were coded by S.K. and primary themes were identified. The identified themes were discussed amongst the research team and a final thematic structure was agreed. To represent the data, a number of quotes are provided in a table to illustrate each theme. NVivo (version 1.6.1) software was used to facilitate the coding process.

The qualitative and quantitative findings were integrated into the interpretation and reporting phase of the study [17]. The findings from qualitative and quantitative data were combined to answer the research questions where the qualitative findings provided a deeper understanding of participants’ answers to the survey. In the results, first, the findings from the quantitative analysis are presented, followed by further details and findings from the qualitative data.

Ethics approval was obtained from the Faculty of Social Sciences and Law Research Ethics Committee at the UoB (Ref: 11080).

## Results

Thirty people expressed interest in attending an ASfL session, of which a total of 25 (83%) participated. Two (7%) could not make any of the available dates and three (10%) did not attend on the day of the session.

A total of five ASfL sessions were conducted between June and July 2022. Of these, four were held online and one was face-to-face. One session was held in Chinese (face-to-face) and all others were conducted in English.

In total, 22 participants (88% of total participants) completed the survey and six (24%) agreed to participate in a semi-structured interview. Interviews lasted between 25 and 50 minutes.

The mean age of those who completed the survey was 30.5 years (standard deviation [SD] = 5.8). Socio-demographic information of the participants was only available from those who completed the survey and are shown in Table 1.

**Table 1.** Characteristics of the participants in Academic Spaces for Listening (*n* = 22)

		*n* (%)
Researcher type		
	PGR	17 (77)
	ECR	5 (23)
Sex		
	Female	17 (77)
	Male	5 (23)
Ethnicity		
	White	6 (27)
	Asian/Asian British	14 (64)
	Latin American	1 (4)
	Turkish	1 (4)
Department		
	Health Sciences	12 (54)
	Social Sciences and Law	1 (4)
	Engineering	5 (23)
	Arts	2 (9)
	Life Sciences	1 (4)
	Science	1 (4)
English proficiency		
	Proficient	7 (32)
	Advanced	10 (45)
	Intermediate	5 (23)
Language preference		
	Prefer sessions in first language	5 (23)
	Prefer sessions in English	5 (23)
	Comfortable with either language	9 (41)
	Not applicable	2 (9)

Survey results showed that for most participants (59%, *n* = 13), the session met their expectations, and exceeded expectations for the rest (41%, *n* = 9). Most participants (68%, *n* = 15) were very satisfied with the information they received before the session, 73% (*n* = 16) were very satisfied with the group size, 68% (*n* = 15) were very satisfied with how the session was organized, 68% (*n* = 15) were very satisfied with how the session ended. None were dissatisfied with any aspect of the session.

Participants were asked about their comfort level if other participants were from the same school, faculty or unknown to them. Less than half the participants (45%, *n* = 10) felt very comfortable being with people from the same school and 9% (*n* = 2) were somewhat uncomfortable. Half the participants (50%, *n* = 11) felt very comfortable with other participants being from the same faculty, and the majority (64%, *n* = 14) felt very comfortable if the other participants were unknown to them.

The majority (64%, *n* = 14) very much agreed that they trusted the group to keep what was shared in the session confidential, while 4% (*n* = 1) very much disagreed. The majority (68%, *n* = 15) of survey respondents very much agreed that they will be more aware of how they listen in the future.

With regards to who should co-ordinate sessions, peer-organized was the most selected option (64% n = 14), followed by the Bristol Doctoral College (59%, *n* = 13) and well-being services (50%, *n* = 11).

When participants were asked about their preferred language for ASfL sessions, 41% (*n* = 9) were comfortable in either English or their first language, while 23% (*n* = 5) preferred the session to be in English and 23% (*n* = 5) preferred the session in their first language.

Data showed that participants were interested in attending future ASfL sessions. Half of the participants (*n* = 11) reported that they would be happy to organize their own sessions. Most participants (68%, *n* = 15) would attend another session and 95% (*n* = 21) of participants would encourage others to attend.

The study identified four main themes in relation to feasibility, acceptability and potential impact of ASfL: the ASfL experience; impact of ASfL; potential challenges of ASfL and ASfL in the future. Exemplary quotes that supported each theme and sub-themes are presented in Table 2.

**Table 2.** Table of identified themes, sub-themes and example quotes from interviews with participants of Academic Spaces for Listening (ASfL)

Theme	Sub-theme	Example quotes
The ASfL experience	Motivation for attending the session	‘I’m normally just by myself, so I need some opportunities to talk’.- Interview 1-PGR ‘So maybe we can share our own problem or difficulties to each other, to get support or help from each other’- Interview 3-PGR
	Feelings and expectations during the session	‘This activity is deeper than I expected […] I think everyone actually said more than I thought. Before it, I thought that everyone would just talk about their own things, not about life or other interpersonal topics’- Interview 6-PGR ‘I wasn’t expecting any sort of feedback or guidance or anything in that sense. Just a space to chat’- Interview 4-ECR
	Interpersonal connectedness	‘I think we said in the session the commonality in how we were feeling when we had different situations […] you can be quite, fairly open and then it doesn’t matter cause you’re not gonna see them again’- Interview 4-ECR ‘I feel that everyone is working hard, …, it’s [the feeling] self-motivated and in a relaxed state’- interview 6-PGR
Impact of ASfL	Personal impact	‘I think after coming to the session, after listening to others talk, I think most of the students or most of the participants have the same problem [ … ] it’s really released and relaxed me’.- Interview 3-PGR ‘It teaches me that I should listen to other people more than before’.- Interview 1-PGR
	Acceptability	‘I think embedding that kind of culture and sort of support and this thing within academia rather than as an extra, it’s kind of an important thing’- Interview 5-ECR ‘Researchers are not necessarily working in teams, especially PGR students can be quite isolating, so having a space to connect with other academics and researchers, just to chat for a few minutes at time, see how they’re feeling that day might be very useful [ … ] It would be a vital part of the puzzle piece of, you know, going from official staff development, leadership type stuff to having chats around the, you know water cooler like, there’s a space in between those two things, I think, academic spaces for listening probably can fill’.- Interview 4- ECR
Potential challenges of ASfL		‘Someone might get very upset or then you know or something might suddenly come up for somebody’.- Interview 5-ECR ‘self-selecting, so you end up getting like the same people like me for example. I’m going to engage in this stuff because I’m already engaging in this stuff, people who might benefit from it may not turn up’ - Interview 4-ECR
ASfL in the future		‘We should extend these events in order to let everyone have the opportunity to talk with others, to hear good and bad experiences and grow with that and this is not only for PhD students or MSc students. There are maybe some lecturers that have these kinds of problems as well’- Interview 2-PGR ‘maybe linking up with support services that exist in the university as well […] having some sort of contingency may be in place if something comes up that’s difficult for someone’- Interview 5-ECR

The use of [ … ] indicates that parts of the text are taken out.

Participants’ experiences of ASfL were explored and three sub-themes were identified: motivation for attending; feelings and expectations during the session; interpersonal connectedness. In relation to motivation for attending, participants had heard about the session mostly through their friends (word of mouth). Specific motivators were curiosity about ASfL, having the opportunity to talk, finding friends, curiosity about their peers, sharing problems and difficulties, helping others and receiving help from peers. Feelings and expectations relating to the session expressed by participants were mixed. Some participants expressed being nervous due to different reasons including not having much to say, not knowing others and the setting being new and different to them. Regarding interpersonal connectedness (how participants relate to each other and how that affects them), the data showed that while knowing other participants encouraged some to share more, others felt they could not share personal matters. Furthermore, participants mentioned that having senior members of the department or people whom they do not get along with could discourage them from sharing or even attending the session. Hearing others disclose how they were feeling, and being vulnerable with the group encouraged participants to share more themselves. Almost all participants mentioned that they found they could relate to each other in the session, and they had a lot in common.

Impacts of ASfL were explored and two sub-themes were identified; personal impact and acceptability. Regarding the first sub-theme participants felt relaxed, heard, supported by peers and engaged during the session. In relation to acceptability, ASfL was perceived as a good initiative to enhance research culture. ASfL was also seen as a place for connecting people and providing an opportunity to share and listen, especially since doing research can be isolating. Participants could see ASfL having the potential to fill a gap in social interactions within the university.

The potential challenges of ASfL highlighted by participants included participation being self-selecting, participants treating sessions as a counselling/therapy session, participants sharing experiences that could be upsetting for others and a lack of support for those who may get upset during the session especially if attended online.

In relation to ASfL in the future, participants provided suggestions for future sessions. Some suggestions were regarding the structure of the session and others were regarding the position of ASfL in the university.

Structural suggestions included having a take-home message for all about the importance of listening and having more time slots available.

The suggestions regarding the position of ASfL included opening sessions to a wider population at the UoB including lecturers and professional staff, being linked to well-being services and having a contingency plan in case any participants become upset during the session.

## Discussion

The results of this pilot study suggest that implementing ASfL in the university setting is a feasible and acceptable initiative with potential to benefit PGR and ECR relationships and well-being. Between June and July 2022, we conducted five ASfL sessions and only a small number of those who confirmed their attendance did not attend the sessions (17% dropout). Conducting five sessions and a low dropout rate showed that implementing ASfL is feasible. Further, participants were satisfied with the content and structure of ASfL. They felt comfortable sharing their thoughts and feelings with group members. Participants stated they would attend and organize future sessions and would encourage others to attend ASfL. There was support for the scalability of ASfL by suggestions to widen eligibility to other members of staff at the UoB and increasing the number of sessions. Such findings showed the acceptability of the ASfL.

Previous studies have shown a sense of loneliness and reduced well-being amongst PGRs and ECRs [7,8]. Findings showed that unmet needs, such as sharing thoughts and feelings, finding friends and having a chance to talk, were amongst the reasons to attend ASfL. Sessions met or exceeded the expectations of the participants. Specifically, ASfL was perceived to fill a gap in the social interaction amongst PGRs and ECRs because it provided an informal, non-judgmental and structured setting and promoted active listening and connectedness. These benefits have been shown to potentially improve well-being and reduce the sense of loneliness amongst PGRs and ECRs [18].

Research output is essential for universities’ reputation and their global ranking, increasing work-related pressures for academic researchers [19]. A recent systematic review and qualitative meta-synthesis suggested that academic researchers experience a high amount of stress mostly caused by trying to meet the performance expectations set by the university [6]. The review showed that researchers are reluctant to share their difficulties as this could have a negative impact on their reputation and subsequently their job security. These findings were similar amongst academics at different career stages. Such studies highlight the need for well-being support as well as creating a healthy workplace by the universities. Our findings showed that most participants felt they could relate to each other and experienced similar feelings. Moreover, at the end of the session, participants felt supported, relaxed and heard. Participants felt that ASfL could be a place to connect to other researchers and university staff, and we know that connecting with others improves well-being [18].

A recent scoping review found a positive impact of peer support in young adults at a university. Peer support delivered in a 1:1 session was associated with increased happiness, self-esteem and effective coping, and decreased depression, loneliness and anxiety. Peer support delivered as a group session was associated with increased well-being and reduced symptoms of depression and anxiety [10]. Our study echoes these findings. Participants in this study were on average 30 years old and equally experienced positive impacts such as relational awareness, reduced sense of loneliness and the importance of active listening. This suggests that routine implementation of peer support initiatives can have wider benefits to a larger staff base.

Practical implications for wider implementation of ASfL sessions in future were identified. First, results suggest that researchers with existing professional relationships (e.g. a more senior member of the research team) attending the same ASfL session could prevent them from freely sharing their thoughts and feelings. Organizing different sessions for researchers at varying levels of seniority may avoid reluctance to disclose difficulties. Second, the study sample was diverse and included participants with varying levels of English language proficiency. It was therefore not surprising that some participants (23%, *n* = 5) preferred to conduct sessions in their first language. Academic institutions commonly comprise a large community of international staff and offering ASfL in different languages can remove barriers to open communication [20,21].

This pilot study had a number of strengths. This study is the first study to evaluate ’Spaces for Listening’, and more specifically in an academic setting. We used a mixed-methods study design that enabled a more in-depth exploration of the findings from the quantitative survey through semi-structured interviews. A further strength of this study is the consideration of participants’ preferences regarding the language. This study adds to the existing evidence on peer support in an academic setting with a specific focus on PGRs and ECRs where the literature is limited. Limitations of this study should be noted. The study was a small pilot study to explore initial indicators of feasibility and acceptability and, as such, did not include standard assessments for well-being and isolation. Future studies may adopt comparative study designs and validated measurement instruments in larger samples to explore the effectiveness of ASfL sessions.

In conclusion, this study showed that implementing ASfL in a university setting was feasible and well received by participants and may help to improve relational awareness, social connection and address feelings of loneliness.

## Supplementary Material

kqae091_suppl_Supplementary_Tables_S1

kqae091_suppl_Supplementary_Tables_S2

## References

[1] Del Cerro Santamaría G. Challenges and drawbacks in the marketisation of higher education within neoliberalism. Rev Eur Stud 2020;12:22.

[2] Kinman G , Johnson S. Special section on well-being in academic employees. Int J Stress Manag 2019;26:159–161.

[3] Kinman G. Doing more with less? Work and wellbeing in academics. Somatechnics 2014;4:219–235.

[4] Fetherston C , Fetherston A, Batt S, Sully M, Wei R. Wellbeing and work-life merge in Australian and UK academics. Stud High Educ. 2020;46:2774–2788.

[5] Wray S , Kinman G. The challenges of COVID-19 for the well-being of academic staff. Occup Med (Lond) 2022;72:2–3. 33585914 10.1093/occmed/kqab007 PMC7928699

[6] Nicholls H , Nicholls M, Tekin S, Lamb D, Billings J. The impact of working in academia on researchers’ mental health and well-being: a systematic review and qualitative meta-synthesis. PLoS One 2022;17:e0268890. 35613147 10.1371/journal.pone.0268890 PMC9132292

[7] Belkhir M , Brouard M, Brunk KH et al. Isolation in globalizing academic fields: a collaborative autoethnography of early career researchers. Acad Manag Learn. Educ 2019;18:261–285.

[8] Jandrić P. Alone-time and loneliness in the academia. Postdigital Sci Educ 2022;4:633–642.

[9] Moak SC , Leban L, Reuter TK. Reentry during a pandemic: a pilot study of access to peer support through technology to reduce social isolation. Am J Crim Justice 2023;48:1204–1223. 10.1007/s12103-022-09690-9 PMC9490686 36159627

[10] Richard J , Rebinsky R, Suresh R et al. Scoping review to evaluate the effects of peer support on the mental health of young adults. BMJ Open 2022;12:e061336. 10.1136/bmjopen-2022-061336 PMC9358944 35926986

[11] Worsley DJ , Pennington A, Corcoran R. Supporting mental health and wellbeing of university and college students: a systematic review of review-level evidence of interventions. PLoS One 2022;17:e0266725. 35905058 10.1371/journal.pone.0266725 PMC9337666

[12] Russell B , Jones C. Spaces for Listening [Internet]. 2020 [cited 1 March 2023]. https://brigidrussell43.medium.com/spaces-for-listening-8c597c1f52a2#_edn2.

[13] NHS Grampian. Spaces for Listening [Internet]. Scotland: NHS Grampian 2022 [cited 1 February 2023]. https://www.nhsgrampian.org/your-health/wecare/noticeboard/2022/february/spaces-for-listening/.

[14] Jones C , Russell B, Yau K-C. Creating Spaces for Listening – What Does it Mean and What Does it Take? By Charlie Jones, Brigid Russell, and King-Chi Yau – The Official Blog of BMJ Leader [Internet]. 2023 [cited 24 June 2024]. https://blogs.bmj.com/bmjleader/2023/11/10/creating-spaces-for-listening-what-does-it-mean-and-what-does-it-take-by-charlie-jones-brigid-russell-and-king-chi-yau/.

[15] Likert R. A technique for the measurement of attitudes. Arch Sci Psychol 1932;22:55.

[16] Braun V , Clarke V. Using thematic analysis in psychology. Qual Res Psychol 2006;3:77–101.

[17] Fetters MD , Curry LA, Creswell JW. Achieving integration in mixed methods designs—principles and practices. Health Serv Res 2013;48:2134–2156. 24279835 10.1111/1475-6773.12117 PMC4097839

[18] NHS—Guides Tools and Activities. 5 Steps to Mental Wellbeing [Internet]. 2022 [cited 1 February 2023]. https://www.nhs.uk/mental-health/self-help/guides-tools-and-activities/five-steps-to-mental-wellbeing/.

[19] Tapper T , Filippakou O. The world‐class league tables and the sustaining of international reputations in higher education. J Higher Educ Pol Manage 2009;31:55–66.

[20] Paloma V , de la Morena I, Sladkova J, López-Torres C. A peer support and peer mentoring approach to enhancing resilience and empowerment among refugees settled in southern Spain. J Community Psychol 2020;48:1438–1451. 32134511 10.1002/jcop.22338

[21] Cherrington AL , Willig AL, Agne AA, Fowler MC, Dutton GR, Scarinci IC. Development of a theory-based, peer support intervention to promote weight loss among Latina immigrants. BMC Obes 2015;2:1–9. 26217532 10.1186/s40608-015-0047-3 PMC4511020

