# Supplementary material for: A novel approach to peer support for academic researchers
Source: Occup Med (Lond). 2024 Oct 19;74(8):601–6. doi: 10.1093/occmed/kqae091 (PMC11604121; doi:10.1093/occmed/kqae091)
Supplement: kqae091_suppl_Supplementary_Tables_S1 [file kqae091_suppl_supplementary_tables_s1.docx]

Supplementary Table S1- Survey questionnaire

| **Section 1: Experience** |  |  |
| --- | --- | --- |
| Are you a PGR (PhD student) or ECR? | Numeric | Multiple choice – PGR, ECR |
| Did you attend an online or in-person session? | Numeric | Multiple choice – online, in-person |
| How satisfied were you with:   1. the information you received before the session 2. the group size 3. how the session was organised 4. how the session ended | Numeric | Likert-scale |
| How much do you agree with the following statements:   1. I felt heard and respected 2. I felt comfortable talking openly with the group 3. I trust that the group will keep what was shared in the session confidential 4. I will be more aware of how I am listening in the future | Numeric | Likert-scale |
| Where your expectations met? | Numeric | Multiple choice – did not meet expectations/ met expectations/ exceeded expectations |
| Would you take part in another session?   - Why? - If yes, would you prefer to continue with the same group or attend with different participants? | Numeric | Yes/No/Unsure   - Multiple choice with OTHER option - Multiple choice |
| Would you encourage other people to take part in the session?   - Why? | Numeric | Yes/No/Unsure   - Multiple choice with OTHER option |
| Would you be interested in facilitating your own session, with support?   - Why? | Numeric | Yes/No/Unsure   - Multiple choice with OTHER option |
| **Section 2: In the future…** |  |  |
| Who do you think should co-ordinate these activities? | Numeric | Multiple choice – peer organised, representatives, Bristol Clear, Bristol Doctoral College, wellbeing services |
| How comfortable would you be if other participants were:   1. Part of the same School 2. Part of the same Faculty 3. Part of the wider University but unknown to you? | Numeric | Likert Scale |

Supplementary Table S1- Survey questionnaire continued

| **Section 3: About you** |  |  |
| --- | --- | --- |
| The questions below are being asked to ensure a range of voices are heard. | | |
| -How is your English proficiency?  -Would you prefer the session in your native language more? (If English is not your first language) | Numeric | 5-point Likert Scale (proficient, advanced, intermediate, elementary, beginner)  Yes/No/ n/a |
| What is your gender? | Numeric | Multiple choice |
| What is your ethnicity? | Numeric | Multiple choice |
| What is your age? | Numeric | Multiple choice |
| Do you identify as having a disability? | Numeric | Multiple choice |
| What Faculty are you from? | Numeric | Multiple choice |
| **Section 4: Final comments** |  |  |
| If you had two words to describe your experience, what would they be? | Open | Free text (limited) |
| Is there anything else you would like to share about your experience? | Open | Free text |
| Would you be happy to take part in a short interview about your experience?   - No: Next question - Yes/Maybe: Links to separate survey where e-mail is entered. | Numeric | Yes/No/Maybe |
