# Supplementary material for: A novel approach to peer support for academic researchers
Source: Occup Med (Lond). 2024 Oct 19;74(8):601–6. doi: 10.1093/occmed/kqae091 (PMC11604121; doi:10.1093/occmed/kqae091)
Supplement: kqae091_suppl_Supplementary_Tables_S2 [file kqae091_suppl_supplementary_tables_s2.docx]

# Supplementary Table S2- Topic Guide for semi-structured interviews

| **Intro to interview** | - Thank you and intro to interviewee and project - Reminder of anonymity - Reminder of withdrawal process - Consent form - Reminder that the participant can decide not to answer any question they are not comfortable with - Ask if there are any more questions before starting the interview - Consent to audio record **** Switch ON recorder **** - Aim/outline of the interview |  |
| --- | --- | --- |
|  | **Questions** | **Prompts** |
| **Experience before the session** | Can you tell me about how you came to attend the session?  How did you feel before attending? What expectations did you have? | How did you find out about the session?  Can you explain why you expected this? |
| **Experience during the session** | Tell me about your experience of participating in the session. | That’s interesting, can you tell me more about that? |
| **Experience after the session** | Please could you describe your reflections following your participation in the session? | After the session ended, what did you take away from your Spaces for Listening experience?  Do you feel it impacted: well-being; peer-to-peer support; listening skills etc  What else did you feel/went through your head following the session? |
| **Looking forward** | *We are running the Spaces for Listening project as part of a wider initiative to enhance research culture.*  How do you feel about the research culture we work in?  What is your view on Spaces for Listening as an initiative to enhance research culture?  How might Academic Spaces for Listening work, or not work, for the university community going forward? | Is it something you feel aware of either in a positive or negative way?  Do you feel that Spaces for Listening could have a role to play in improving research culture or the experiences of researchers/postgraduate researchers?  What do you feel needs to change/happen for Spaces for Listening to run in this way at the University?  What changes do you suggest might need to be made to encourage people to take part? |
| **Summary and end of session** | - Summarise key points from interview - Check in with participant on how they are doing - Ask if anything participant wishes to add - Thank participant and end interview **** Switch OFF recorder **** |  |
